# Supplementary material for: Immunofocusing and enhancing autologous Tier-2 HIV-1 neutralization by displaying Env trimers on two-component protein nanoparticles
Source: NPJ Vaccines. 2021 Feb 9;6:24. doi: 10.1038/s41541-021-00285-9 (PMC7873233; doi:10.1038/s41541-021-00285-9)
Supplement: Supplementary file 1 — Supplementary Information [file 41541_2021_285_MOESM1_ESM.pdf]

Supplementary Information

Immunofocusing and enhancing autologous Tier-2 HIV-1 neutralization by displaying Env trimers on two-component protein nanoparticles

Philip J. M. Brouwer[1], Aleksandar Antanasijevic[2], Marlon de Gast[1], Joel D. Allen[3], Tom P. L. Bijl[1], Anila Yasmeen[4], Rashmi Ravichandran[5,6], Judith A. Burger[1], Gabriel Ozorowski[2], Jonathan L. Torres[2], Celia LaBranche[7], David C. Montefiori[7], Rajesh P. Ringe[4,¥], Marit J. van Gils[1], John P. Moore[4], Per Johan Klasse[4], Max Crispin[3], Neil P. King[5,6,*], Andrew B. Ward[2,*], Rogier W. Sanders[1,4*]

Affiliations:

[1]Department of Medical Microbiology, Amsterdam UMC, University of Amsterdam, Amsterdam Infection & Immunity Institute, 1105AZ Amsterdam, The Netherlands

[2]Department of Integrative Structural and Computational Biology, The Scripps Research Institute, La Jolla, CA 92037, USA

[3]School of Biological Sciences, University of Southampton, Southampton SO17 1BJ, UK

[4]Department of Microbiology and Immunology, Weill Medical College of Cornell University, New York, NY 10065, USA

[5]Department of Biochemistry, University of Washington, Seattle, WA 98195, USA

[6]Institute for Protein Design, University of Washington, Seattle, WA 98195, USA

[7]Department of Surgery, Duke University Medical Center, Durham, NC 27710, USA

[¥]Present address: Institute of Microbial Technology, Chandigarh, 160036, India

[*]Corresponding author: r.w.sanders@amsterdamumc.nl; andrew@scripps.edu; neil@ipd.uw.edu

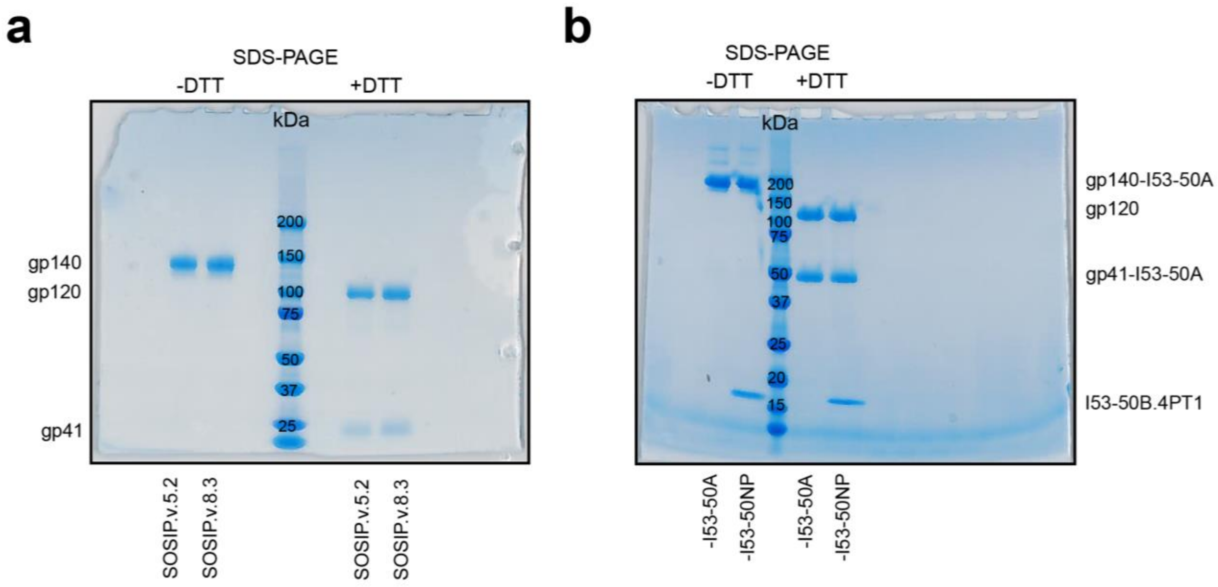

Supplementary Fig. 1. Biophysical characterization of 16055 SOSIPs, 16055 SOSIP-I53-50A, and 16055 SOSIP-I53-50NP **a** Non-reducing (-DTT) and reducing (+DTT) SDS-PAGE of 16055 SOSIP.v5.2 and 16055 SOSIP.v8.3. **b** As panel-a but for 16055 SOSIP-I53-50A and 16055 SOSIP-I53-50NP.

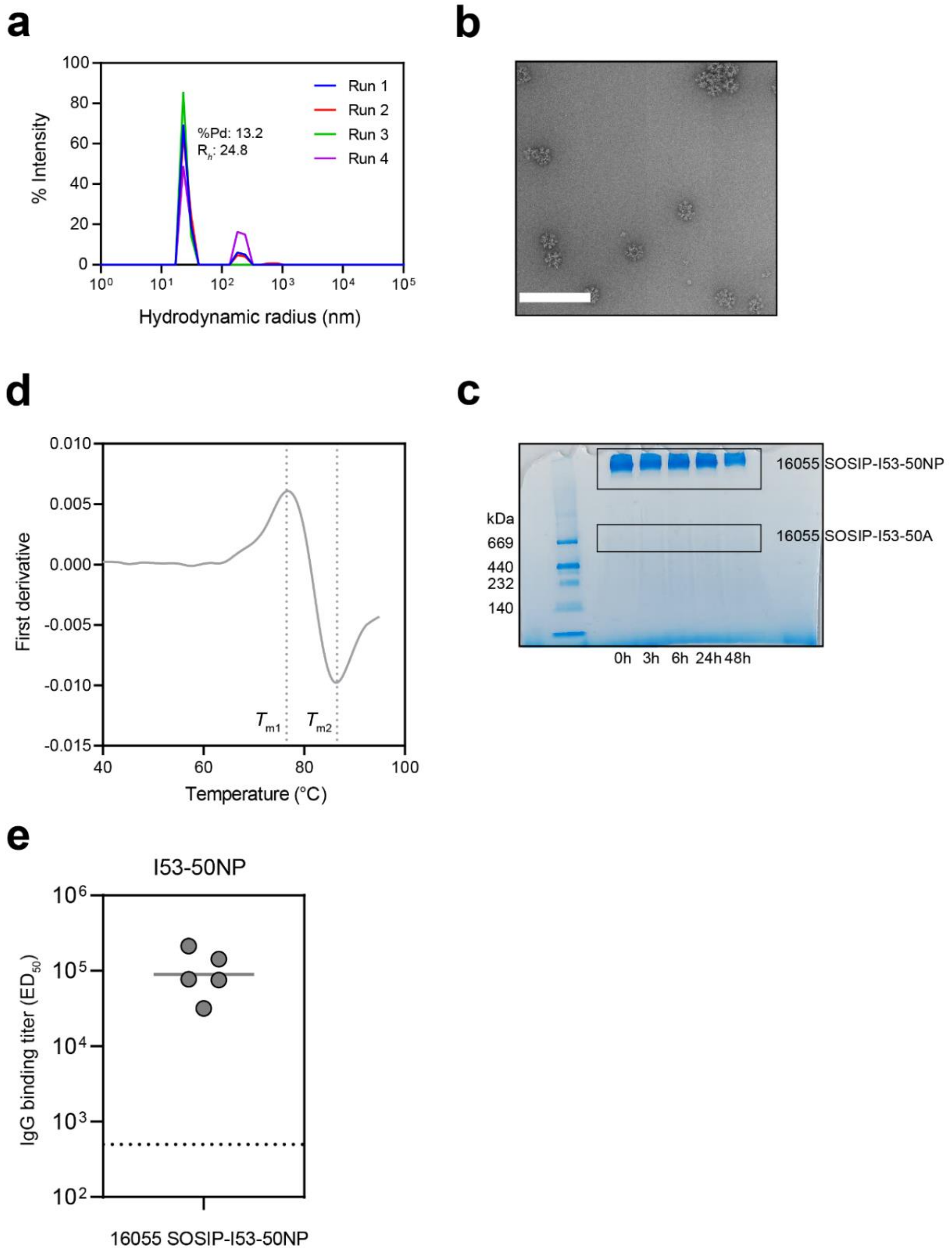

Supplementary Fig. 2. Biophysical characterization of 16055 SOSIP-I53-50NPs and the immunogenicity of the I53-50NP core. **a** Dynamic light scattering data of 16055 SOSIP-I53-50NPs. Average polydispersity (%Pd) and hydrodynamic radius (R_h) are shown for the non-aggregated 16055 SOSIP-I53-50NP population. A %Pd < 15 is considered a monodisperse population. An overlay of four individual runs is shown. **b** Raw nsEM micrograph of 16055 SOSIP-I53-50NPs showing the small percentage of aggregates. White scale bar corresponds to 200 nm. **c** Thermostability of 16055 SOSIP-I53-50NP as determined by NanoDSF. The dotted lines indicate the melting temperatures of the 16055 SOSIP (T_{m1}) and I53-50NP core (T_{m2}). Representative melting curve of three technical replicates is shown. **d** Native-PAGE of 16055 SOSIP-I53-50NP incubated at 37°C in PBS for 0, 3, 6, 24 and 48 h. The upper box indicates the band for 16055 SOSIP-I53-50NPs whereas the lower indicates where a 16055

SOSIP-I53-50A band would have been visible upon nanoparticle disassembly. **e** Midpoint binding titers against I53-50NPs from week 22 rabbits sera of 16055 SOSIP-I35-50NP recipients. Horizontal bar indicates the geometric mean. The dotted line indicates the lowest dilution.

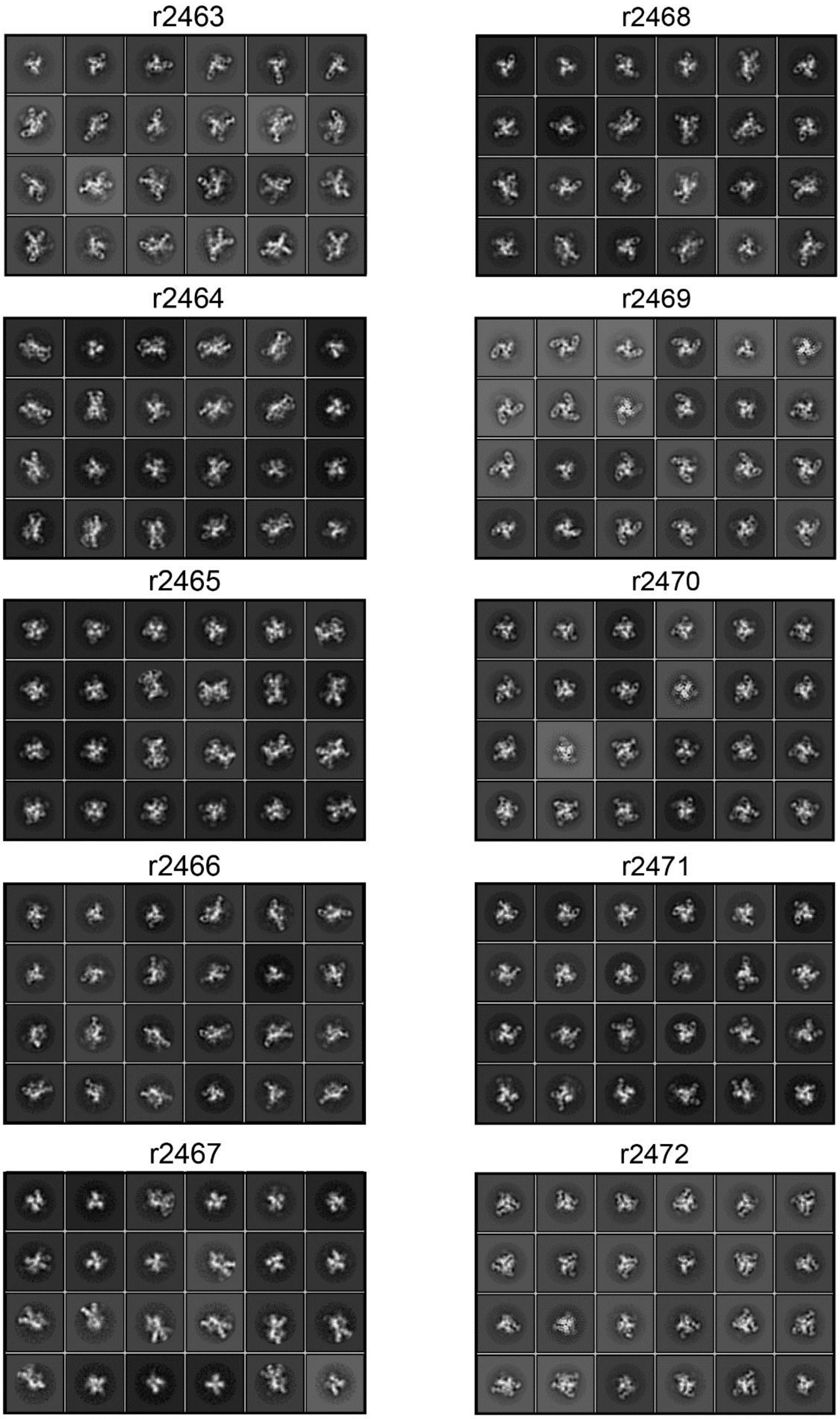

Supplementary Fig.3. Representative 2D class averages of 16055 SOSIP trimers complexed with polyclonal Fabs from rabbit sera with IDs indicated above.

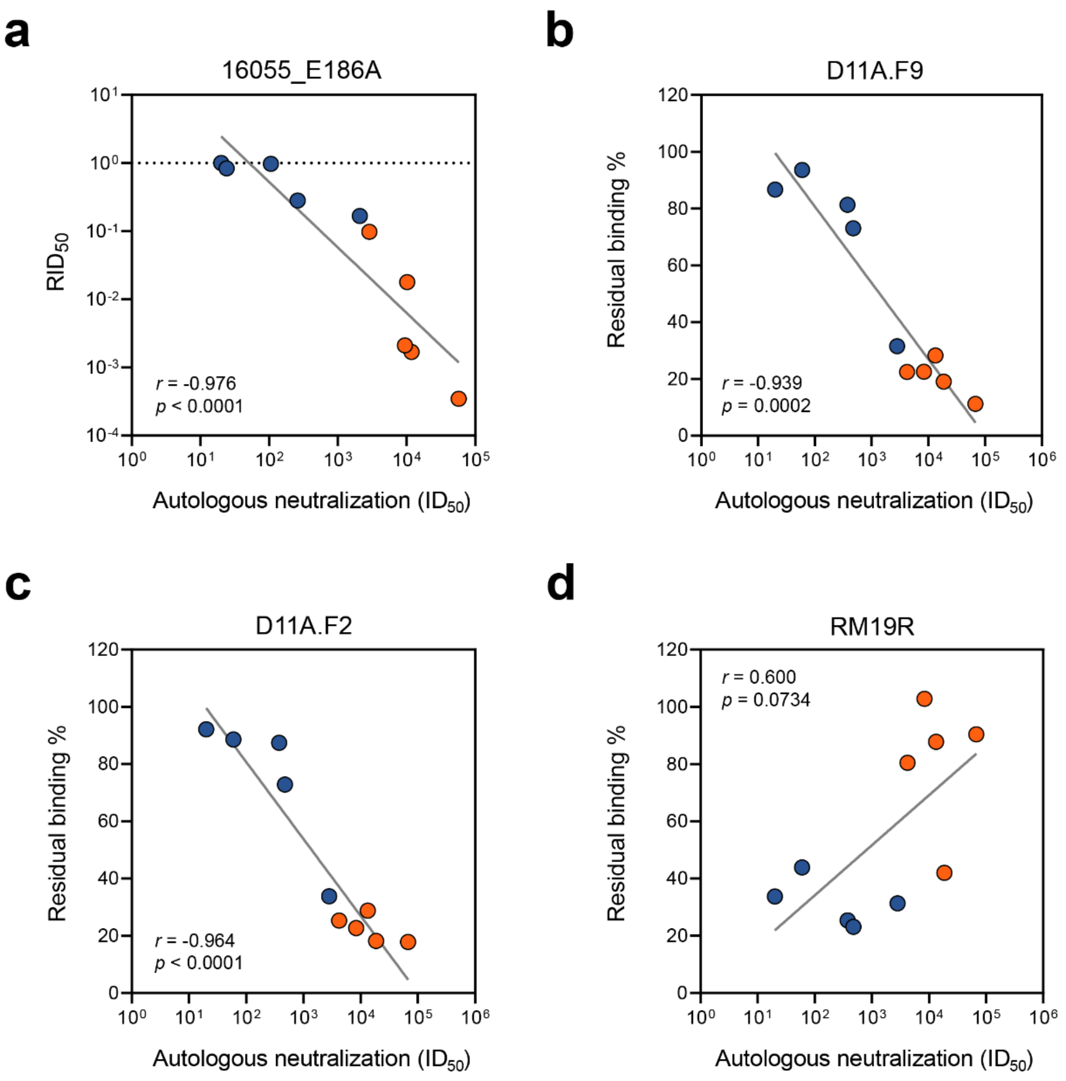

Supplementary Fig. 4. Epitope mapping of NAb responses in rabbits immunized with 16055 SOSIP or 16055 SOSIP-I53-50NPs. **a** Correlation plot of RID50 versus 16055 midpoint neutralization titers. **b** Correlation plot of D11A.F9 competition versus 16055 midpoint neutralization titers. **c** The same as b but then for D11A.F2 **d** The same as b but then for RM19R. **a-d** The *r* and *p* value is shown for non-parametirc Spearman rank correlation. Blue and orange symbols represent individual rabbits immunized with 16055 SOSIP or 16055 SOSIP-I53-50NPs, respectively.

	Mutation	SOSIP nomenclature					Reference
		v4	v5	v8.1	v8.2	v8.3	
	501C-605C						Sanders et al., Plos Pathogens 2013
	559P						
	R6						
	.664						
	448N						
	64K/66R[a]						de Taeye et al., Cell 2015; Dey et al., Virology 2008
	315Q						
	316W						
	535M[b]						
	543N/543Q[c]						
	73C-561C[d]						Torrents de la Pena et al., Cell Reports 2017
TD8	47D						Guenaga et al., Journal of Virology 2016
	49E						
	65K						
	106T						
	165L						
	429R						
	432Q						
	500R						
MD39	106E						Steichen et al., Immunity 2017
	271I						
	288L						
	304V						
	319Y						
	363Q[e]						
	519S						
	561P[f]						
	568D						
	570H						
	585H						
	569G						Guenaga et al., Immunity 2017

Supplementary Table 1. Overview of mutations that make up SOSIP v4-v8.3.

[a] The variant 64K (v4.1) is used for BG505 and B41; while the variant 66R (v4.2) is used for the rest of strains.

[b] This mutation was not introduced in 16055 SOSIP.

[c] de Taeye et al. identified two variants (543N and 543Q) with positive effects. 16055 naturally contains a Q at position 543.

[d] 72C-564C (v5.1) is an alternative disulfide bond that works as well as 73C-561C (v5.2), but we generally work with the latter.

[e] Is left out in several genotypes (BG505) as it introduces a glycan hole. This is not the case for 16055.

[f] Mutation 561P (Steichen et al, Immunity 2017) is not included, as we routinely introduce a Cys in this position for the v5 disulfide bond.

16055 SOSIP

	88	160	187	197	262	276	289	301	360	386	409	442	448	463	611	616	625	637
High Mannose	41	65	56	34	100	90	100	62	100	100	49	93	100	43	0	1	35	84
M9	0	1	0	0	83	0	68	0	68	78	0	17	5	0	0	0	0	0
M8	0	11	0	13	14	7	28	0	20	20	0	67	67	0	0	0	0	0
M7	0	3	0	13	1	1	4	0	10	2	0	1	14	0	0	1	5	17
M6	8	19	0	4	1	43	0	0	1	0	0	4	8	0	0	0	0	24
M5	27	28	54	0	0	33	0	62	0	0	49	3	5	39	0	0	29	32
M4	0	1	0	0	0	1	0	0	1	0	0	0	1	0	0	0	0	3
M3	0	0	0	0	0	0	0	0	0	0	0	0	0	0	0	0	0	0
Hybrid	5	1	0	2	0	2	0	0	0	0	0	0	0	2	0	0	0	7
FHybrid	0	0	2	1	0	0	0	0	0	0	0	0	0	2	0	0	0	0
HexNAC	1	0	0	1	0	1	0	0	0	0	0	0	0	1	0	0	0	1
A1	10	1	2	0	0	2	0	0	0	0	0	0	0	0			4	6
FA1	0	0	2	7	0	0	0	35	0	0	39	0	0	10			3	3
A2/A1B	1	0	1	0	0	1	0	0	0	0	0	0	0	0			7	0
FA2/FA1B	17	1	36	3	0	0	0	0	0	0	13	0	0	41	0	0	36	2
A3/A2B	4	0	0	0	0	0	0	0	0	0	0	0	0	0			0	0
FA3/FA2B	19	0	3	0	0	0	0	0	0	0	0	0	0	4			11	2
A4/A3B	0	0	0	0	0	0	0	0	0	0	0	0	0	0			0	0
FA4/FA3B	0	0	0	0	0	0	0	0	0	0	0	0	0	1			0	0
Unoccupied	6	34	1	56	0	7	0	3	0	0	0	7	0	0	0	60	3	2

	88	136	139	145	156	160	187	197	230	234	241	262	276	289	301	360	386	392	399	404	409	442	448	463	611	616	625	637
High Mannose	42	69	69	0	63	65	56	34	35	100	94	100	90	100	62	100	100			0	49	93	100	43	8	1	35	84
Complex	52	18	31	100	1	2	43	11	0	0	0	0	3	0	35	0	0			100	51	0	0	57	63	39	62	14
Unoccupied	6	14	0	0	36	34	1	56	65	0	6	0	7	0	3	0	0			0	0	7	0	0	29	60	3	2

16055 SOSIP-I53-50A

	88	145	156	160	187	197	234	241	262	276	289	301	360	386	409	442	448	463	625	637
High Mannose	69	58	44	84	56	66	100	0	100	97	100	82	99	100	42	97	98	16	86	93
M9	0	0	23	0	0	0	76	100	89	1	18	7	74	80	0	73	0	0	0	0
M8	0	0	17	0	0	25	24	0	11	13	58	0	20	18	0	4	87	0	0	17
M7	0	4	0	12	0	12	0	0	0	11	25	0	1	2	7	0	0	0	1	40
M6	30	0	1	29	13	0	0	0	1	43	0	6	0	1	2	7	7	9	0	5
M5	33	46	3	39	41	17	0	0	0	26	0	64	1	0	33	11	2	1	84	28
M4	1	0	0	0	1	0	0	0	0	1	0	0	0	0	0	0	1	0	0	1
M3	0	0	0	0	0	0	0	0	0	0	0	0	0	0	0	0	0	0	0	0
Hybrid	3	0	0	2	0	6	0	0	0	0	0	0	1	0	0	0	0	0	1	0
FHybrid	0	0	0	0	0	0	0	0	0	0	0	0	0	0	0	0	0	1	0	0
HexNAc	2	9	0	2	1	6	0	0	0	1	0	5	1	0	0	2	0	5	0	3
A1	8	0	0	1	3	0	0	0	0	2	0	0	0	0	0	0	2	4	0	0
FA1	0	5	0	0	0	0	0	0	0	0	0	0	0	0	0	1	0	40	0	2
A2/A1B	12	0	0	0	0	1	0	0	0	0	0	0	0	0	0	0	0	5	7	0
FA2/FA1B	0	4	0	0	40	13	0	0	0	0	0	0	0	0	0	0	0	24	1	4
A3/A2B	3	0	0	0	0	0	0	0	0	0	0	0	0	0	0	0	0	0	1	0
FA3/FA2B	7	0	0	0	1	3	0	0	0	0	0	0	0	0	20	0	0	8	4	1
A4/A3B	0	0	0	0	0	0	0	0	0	0	0	0	0	0	0	0	0	0	0	0
FA4/FA3B	0	0	0	0	0	0	0	0	0	0	0	0	0	0	0	0	0	4	0	0
Unoccupied	0	32	56	15	0	17	0	0	0	1	0	18	1	0	37	2	0	0	0	0

	88	136	139	145	156	160	187	197	230	234	241	262	276	289	301	360	386	392	399	404	409	442	448	463	611	616	625	637
High Mannose	69	97	93	58	44	84	56	66	n.d.	100	100	100	97	100	82	99	100	n.d.	n.d.	0	42	97	98	16	41	14	86	93
Complex	30	0	0	10	0	1	44	17	n.d.	0	0	0	3	0	0	0	0	n.d.	n.d.	100	20	2	2	84	42	44	13	7
Unoccupied	0	3	7	32	56	15	0	17	n.d.	0	0	0	1	0	18	1	0	n.d.	n.d.	0	37	2	0	0	16	42	0	0

Supplementary Table 2. Glycan composition of 16055 SOSIP and 16055 SOSIP-I53-50A. Glycoforms are shown for each PNGS of 16055 SOSIP (top) and 16055 SOSIP-I53-50A (bottom), with each number representing the percentage of the specific glycoform as stated in the left column. Oligomannos/hybrid-type glycans are shaded in green and complex glycans are shaded in pink. The absence of a glycan is shaded in grey. Glycoforms are categorized according to the number of mannose residues (M3-M9), hybrid glycans by the precense/absence of fucose (FHybrid and Hybrid), and complex glycans by the presence/absence of fucose and the number of antenna. The upper table for each construct contains a breakdown of compositions obtained using intact glycopeptides. The percentages in lower table are obtained by using glycosidase-treated peptides (see Methods for details). Sites that appear in the lower table but not the upper are those that could not be obtained

Week 6

Immunogen		Control		Autologous	
	Virus	MLV		16055	
	Tier	-		2	
	Clade	-		C	
	Lab	AMC	DUMC	AMC	DUMC
Immunogen	Rabbit ID				
16055 SOSIP	2463	27	n.d.	20	n.d.
	2464	21	n.d.	<20	n.d.
	2465	54	n.d.	92	n.d.
	2466	<20	n.d.	<20	n.d.
	2467	<20	n.d.	<20	n.d.
16055 SOSIP-I53-50NP	2468	<20	n.d.	87	n.d.
	2469	22	n.d.	55	n.d.
	2470	<20	n.d.	<20	n.d.
	2471	20	n.d.	92	n.d.
	2472	22	n.d.	26	n.d.

Week 22

		Control		Autologous		Heterologous	
	Virus	MLV		16055		SF162	MW965
	Tier	-		2		1A	1A
	Clade	-		C		B	C
	Lab	AMC	DUMC	AMC	DUMC	AMC	DUMC
Immunogen	Rabbit ID						
16055 SOSIP	2463	<20	<20	20	20	123	1672
	2464	<20	<20	2814	1793	<20	837
	2465	<20	<20	376	158	<20	348
	2466	<20	<20	60	25	<20	108
	2467	<20	<20	474	186	<20	212
16055 SOSIP-I53-50NP	2468	<20	<20	18660	4870	<20	479
	2469	<20	<20	13348	4333	<20	109
	2470	<20	<20	8363	2923	<20	3585
	2471	<20	<20	67677	26174	<20	2000
	2472	<20	<20	4200	1550	<20	563

Supplementary Table 3. Midpoint neutralization titers at week 6 and 22 from rabbits that received 16055 SOSIP or 16055 SOSIP-I53-50NP tested against a panel of Env-pseudoviruses. TZM-bl neutralization assays were performed either at the AMC and/or DUMC as indicated above each column. ID_{50} values, i.e. the serum dilution at which infectivity was inhibited by 50%, are shown and color coded: white = no neutralization, ID_{50} < 20; grey = very weak neutralization, 20 > ID_{50} >40; yellow = weak neutralization, 40 > ID_{50} > 100; orange = moderate neutralization, 100 > ID_{50} > 1000; red = strong neutralization, 1000 > ID_{50} > 10000; purple = very strong neutralization, ID_{50} > 10000. MLV = murine leukemia virus (negative control). n.d. = not determined.

Week 22

		Heterologous								
	Virus	25710-2.43	TRO.11	BJOX002000.03.2	Ce1176_A3	X1632_S2_B10	246-F3_C10_2	CH119.10	Ce703010217_B6	CNE55
	Tier	2	2	2	2	2	2	2	2	2
	Clade									
	Lab	DUMC	DUMC	DUMC	DUMC	DUMC	DUMC	DUMC	DUMC	DUMC
Immunogen	Rabbit ID									
16055 SOSIP	2463	<20	<20	<20	<20	<20	<20	<20	<20	<20
	2464	<20	<20	<20	<20	<20	<20	25	<20	<20
	2465	<20	<20	<20	51	<20	28	44	<20	21
	2466	<20	<20	<20	<20	<20	<20	21	<20	<20
	2467	<20	<20	<20	<20	<20	<20	<20	<20	<20
16055 SOSIP-I53-50NP	2468	<20	<20	<20	28	<20	<20	34	<20	<20
	2469	<20	<20	<20	<20	<20	<20	<20	<20	<20
	2470	<20	<20	<20	<20	<20	<20	<20	<20	<20
	2471	<20	<20	<20	<20	<20	<20	<20	<20	<20
	2472	<20	<20	<20	40	<20	<20	24	<20	<20

Supplementary Table 4. Midpoint neutralization titers at week 22 from rabbits that received 16055 SOSIP or 16055 SOSIP-I53-50NP tested against a panel of heterologous Env-pseudoviruses. TZM-bl neutralization assays were performed at DUMC as indicated above each column. ID_{50} values, i.e. the serum dilution at which infectivity was inhibited by 50%, are shown and color coded as shown in the legend for Supplementary Table 3.
